# Associations between adverse childhood family environments and blood pressure differ between men and women

**DOI:** 10.1371/journal.pone.0225544

**Published:** 2019-12-04

**Authors:** Hannah M. C. Schreier, Emily J. Jones, Sibel Nayman, Joshua M. Smyth

**Affiliations:** 1 Department of Biobehavioral Health, The Pennsylvania State University, University Park, PA, United States of America; 2 Department of Psychology, University of Mannheim, Mannheim, Germany; 3 Center of Psychological Psychotherapy, Mannheim, Germany; University of Jyvaskyla, FINLAND

## Abstract

**Background:**

It is unclear how adverse childhood family environments differentially impact adult health outcomes among men and women. This brief communication reports on the independent and joint effects of adverse childhood family environments and sex on indicators of health in adulthood.

**Methods & Results:**

213 18-55-year olds reported on their childhood family environment (Risky Families Questionnaire (RFQ); Family Environment Scale (FES_total_)) and their current perceived stress and depressive and anxious affect. Resting systolic (SBP) and diastolic blood pressure (DBP), and heart rate (HR) were taken during a laboratory visit, and total cortisol output was measured in saliva samples collected at home. Exposure to childhood adversity did not vary by sex. Women had lower SBP, DBP, and total cortisol output, but higher HR, than men (*p*s < .05). Sex moderated the association between childhood family environment and SBP (RFQ: B = -.316; SE = .120; *p* = .009; FES_total_: B = -.274; SE = .117; *p* = .021) and DBP (FES_total:_ B = -.193; SE = .094; *p* = .041), such that exposure to greater childhood adversity was linked to lower BP in women only. Results were largely unchanged after adjusting for concurrent perceived stress and depressive and anxious affect. Separate effects of individual FES subscales are also discussed.

**Conclusions:**

Contrary to expectations, exposure to adverse childhood family environments was associated with lower resting BP among women, perhaps indicative of basal cardiovascular hypoactivation, whereas early adversity was not linked to BP among men.

## Introduction

Experiences of early adversity, including growing up in a harsh family environment or experiencing child maltreatment, have been linked to adverse health outcomes later in life [1,2], including cardiovascular disease (CVD)[3,4]. Although such exposures are generally considered to increase the likelihood of later health problems, there is some evidence to suggest that men and women may be differentially impacted by such experiences [5–7]. For example, greater cardiovascular reactivity to and slower recovery from acute psychosocial stressors has been reported among young and middle-aged (though not older) women compared to men [8]. Moreover, women may be more vulnerable than men when faced with stressors that are interpersonal in nature or when witnessing people around them experience stressors [9]. Thus, growing up in harsher family environments may be more detrimental for women as this would involve not only experiencing interpersonal stressors oneself, but further bearing witness to conflict and negative interactions among other family members, such as between parents or between parents and siblings. In the long run, such differences in acute stress reactivity and recovery may contribute to different rates of disease, including increased risk for CVD [10].

Two recent reports highlight sex differences following exposure to early adversity, specifically retrospectively self-reported abuse during childhood. Chen et al. [11] found that abuse only predicted later all-cause mortality among women, not men, even though rates of exposure to abuse either did not vary by sex or were higher among men. In addition, the associations between childhood abuse and later all-cause mortality among women were not explained by demographic variables, including socioeconomic status, and personality and affective traits, such as depression. Similarly, Suglia et al. [12] found that hypertension was more prevalent among young women, but not men, who retrospectively reported having experienced childhood sexual abuse. Despite such clear sex differences, the biological and psychological pathways connecting early adversity to different mortality and morbidity outcomes among men and women are not clearly understood; moreover, it is unclear whether similar sex differences are found in response to less severe childhood adversity. Although some previous research has highlighted longitudinal associations between psychosocial variables during childhood [13–15], these studies frequently combine a range of diverse indicators of participants’ early life environment, e.g., parent occupation, parent health behaviors, and the occurrence of specific stressful events (e.g., parental divorce). This makes it difficult to draw conclusions about the relative contributions of these different aspects of participants’ childhood environments on future health-relevant outcomes. The present study expands on this research by focusing in greater depth on the psychosocial family environment, i.e. the overall family climate in particular. Compared to focusing on, e.g., specific, acute stressful life events that people may have experienced during childhood, this likely provides a clearer picture of the quality of everyday interactions in participants’ childhood homes. Moreover, the effect of participant sex is typically adjusted for but not examined separately. Thus, the present study further expands on existing research by directly examining possible moderation by sex.

This study aims to investigate the influence of exposure to moderate early adversity on some physiological indicators, including resting blood pressure (BP) and heart rate (HR) and salivary cortisol that may represent pathways through which exposure to early adversity can lead to morbidity and mortality later in life. Elevated resting BP and HR are known risk factors for CVD [16,17] and dysregulated cortisol production has been implicated in the pathogenesis of CVD, in part through accelerating atherosclerosis [18,19]. To this end, we investigate the influence of a harsher family climate during childhood on the above outcomes, hypothesizing that people exposed to more childhood adversity will have higher BP, HR and cortisol levels, on average. We further investigate the possible moderation of this association by sex. Relative to men, we expect that women may be more strongly impacted, experiencing higher BP, HR, and cortisol levels following exposure to childhood adversity compared to men. Finally, to assess whether possible effects of harsher family environments on adult physiological outcomes may be a function of affective states and/or stress exposure in adulthood, we include measures of participants’ concurrent anxious and depressive affect, as well as perceived stress.

## Materials and methods

### Participants

213 adults (30.1 ± 10.8 years old; 123 men; 66.7% White) involved in the Pittsburgh Common Cold Project 3 (PCS3) were recruited from the greater Pittsburgh area from 2007–2011 via newspaper advertisements. Inclusion criteria were being fluent in English and being in good health as per a physician examination. Participants were excluded if they were currently pregnant, lactating, immunocompromised, diagnosed with and treated for a chronic disease or psychiatric diagnosis within the past year, or taking any regularly prescribed medications other than hormonal birth control. Men and women did not differ in age, education, race/ethnicity, marital status or employment status but women had higher body mass indices (BMI; t(130.86) = -2.246; *p* = .026) and were less likely to be smokers (X^2^(1, N = 213) = 6.100; *p* = .014); Table 1.

**Table 1:** Sample descriptives by participant sex.

	Men (n = 123; 57.7%)		Women (n = 90; 42.3%)	
	n (%)	M (SD)	n (%)	M (SD)
Age (years)		29.84 (10.33)		30.53 (11.57)
Race and Ethnicity				
White/Caucasian	87 (70.7)		55 (61.1)	
Other Race/Ethnicity	36 (29.3)		35 (38.9)	
Years of Education		13.93 (2.00)		
Employed	72 (58.5)		56 (62.2)	
Married/domestic partnership	18 (14.6)		14 (15.6)	
Current Smoker	50 (40.7)		22 (24.4)*	
BMI, kg/m^2^		26.64 (4.68)		28.71 (8.23)*
Childhood Adversity				
RFQ		28.29 (9.96)		28.15 (10.67)
FES Family Relationships (FES_total_)		41.06 (10.16)		42.42 (10.87)
FES_conflict_		14.88 (4.05)		14.64 (4.19)
FES_cohesion_		12.29 (4.27)		13.04 (4.69)
FES_expressiveness_		13.89 (3.40)		14.73 (3.51)
Outcome Measures				
SBP, mm Hg		120.76 (9.53)		112.75 (10.87)*
DBP, mm Hg		73.09 (7.73)		70.47 (7.16)*
HR, bpm		72.21 (10.58)		76.26 (8.86)*
Total Cortisol, AUC; log		3.70 (.20)		3.64 (.23)*
Adult Distress Measures				
Perceived stress		12.28 (5.58)		11.73 (5.76)
Depressive affect		0.89 (1.17)		1.05 (0.92)
Anxious affect		1.22 (1.18)		1.20 (1.05)

Note

* p < 0.05 when comparing men and women using independent sample T-tests and chi-squared tests; BMI = body mass index; RFQ = Risky Families Questionnaire; FES = Family Environment Scale; SBP = systolic blood pressure; DBP = diastolic blood pressure; HR = heart rate. The FES was reverse scored to be in line with the RFQ. Higher scores on the FES family relationship index and subscales are indicative of *more* conflict and *less* cohesion and expressiveness within the childhood home. One female participant who was taking corticosteroid medication was excluded from analyses for total cortisol.

### Procedure

Data were accessed via the Common Cold Project website (www.commoncoldproject.com; grant number NCCIH AT006694). The larger project included the experimental examination of participants’ susceptibility to viral challenge; the analyses reported here use only baseline data collected prior to experimental procedures. Participants provided written consent, completed demographic and psychosocial questionnaires and reported on their daily mood over a two-week period. Participants’ BP, HR, height and weight were taken during a laboratory visit and participants collected saliva samples at home. The study was approved by the institutional review boards at Carnegie Mellon University and The University of Pittsburgh.

### Measures

#### Childhood adversity

Participants completed the 13-item Risky Families Questionnaire (RFQ)[20] assessing how frequently they were exposed to a variety of adverse physical and psychological circumstances in their childhood homes when they were 5–15 years. On a 5-point Likert scale (1 = not at all; 5 = very often), participants noted how often they experienced certain distressing events (e.g., domestic disputes; parental substance abuse) and the extent to which care and affection were provided. Positively-framed items (e.g., being hugged) were reverse scored, with higher total RFQ scores indicative of more adversity. Scores ranged from 13–63 and internal consistency was strong (Cronbach’s α = .90). RFQ scores were comparable to those reported in previous reports [21,22].

Participants also completed the Family Environment Scale (FES)[23], a well-validated 25-item scale about family relationships and family system maintenance when they were 5–15 years. The three subscales making up the family relationships index (conflict; cohesion; expressiveness; 15 items) were deemed relevant to the question at hand and included here. On a 5-point Likert scale (1 = strongly disagree; 5 = strongly agree), participants reported the extent to which they agreed with each statement. To align with the RFQ, positively-framed items were reverse scored so higher scores reflect more conflict and less cohesive and expressive family environments. Internal consistency was acceptable to very good for the overall family relationships index (FES_total_; Cronbach’s α = .89) and across the conflict, cohesion and expressiveness dimensions (Cronbach’s α = .59-.89), which is similar to previously reported values [23].

#### Physiological measures

During day 0 (baseline) of quarantine, resting systolic (SBP) and diastolic (DBP) blood pressure and HR were assessed by study staff three times over the course of one day, i.e., at 8:00 AM, 1:00 PM, and 7:00 PM, using an automated, portable oscillometric blood pressure monitor (Spot Vital Signs, Welsch Allyn, Inc., Skaneateles Falls, NY, USA) and averaged across the three sessions. Height and weight were measured at two laboratory visits using a standard balance scale, BMI (kg/(m^2^)) calculated, and averaged across the two visits. Participants collected saliva samples using Salivettes (Sarstedt, Rommelsdorf, Germany) over 2 days, 7 samples per day across the day (60, 120, 240, 420, 540, 660, and 780 minutes following wakeup). Participants were advised not to eat or brush their teeth one hour before saliva collection, to abstain from smoking 30 minutes prior to collection, and were given a kitchen timer and either a preprogrammed wristwatch or handheld computer to increase compliance. Cortisol levels were determined by time-resolved fluorescence immunoassays with cortisol-biotin conjugates as tracers [24]. Intra- and inter-assay coefficients of variation were < 12%. Total cortisol was estimated by calculating area under the curve (AUC) using the trapezoidal rule [25] for each day and averaging across the two days for each person. Only data from saliva samples taken within 45–90 min. following wakeup (sample 1) or within 60 min. of the prescribed time (all other samples) were included.

#### Adult distress measures

Participants completed the 10-item Perceived Stress Scale (PSS) [26] at a pre-quarantine in-person visit. On a 5-point Likert scale (0 = never; 4 = very often), they indicated how frequently they had experienced certain thoughts and feelings over the past month (Cronbach’s α = .812). Items were summed; higher scores reflect greater perceived stress. During daily, evening phone interviews over 2 weeks, participants reported how often they experienced each of 16 emotions that day on a 5-point Likert Scale (0 = they did not feel that way at all; 4 = frequently feeling that way that day). Mean depressive affect (mean of responses to ‘sadness’ and ‘unhappiness’) and mean anxious affect (mean of responses to being ‘on edge’ and ‘tense’) were calculated.

### Statistical analyses

One participant reported corticosteroid medication use and was excluded from analyses for total cortisol output. Total cortisol output was not normally distributed and log transformed. All models adjusted for race/ethnicity (White vs. Non-White), age, years of education and birth control use. Models regressing BP and HR on early adversity exposure additionally adjusted for BMI. Multiple linear regression models were fit to assess the main effects of the childhood family environment and interaction effects of childhood family environment X sex on physiological outcomes. All covariates and independent variables were first centered at zero and interaction terms were created by multiplying each centered childhood family environment variable (RFQ, FES_total_, FES_cohesion_, FES_expressiveness_, FES_conflict_) by sex (coded as 1 = female; 0 = male). Covariates and independent variables were entered in Step 1 and the interaction term was added in Step 2. When considering adult distress measures related to adult distress/dysphoria, we first examined the independent effect of each, then the effects of all 3 in saturated models. Statistical models were analyzed using SPSS version 24.0 (IBM, New York, NY).

## Results

### Main effects of adversity in childhood family environments and sex

There was a significant, negative effect of FES_conflict_ (B = -.320; SE = .152; *p* = .037) on SBP which remained significant when adding current depressive affect (*p* = .041), but not when adding anxious affect and stress (*ps* > .05). Other aspects of the family environment were not associated with physiological outcomes (all *ps* > .10). Moreover, women had lower SBP (B = -9.856; SE = 1.310; *p* < .001), DBP (B = -3.314; SE = 1.042; *p* = .002) and total cortisol output (B = -.070; SE = .032; *p* = .032), but higher HR (B = 3.043; SE = 1.402; *p* = .031) than men. When including adult distress measures in the models, effects remained significant except when depressive affect was added to models for total cortisol output (*ps* > .05).

### Interaction effects between adversity in childhood family environments and sex

**Fig 1:**
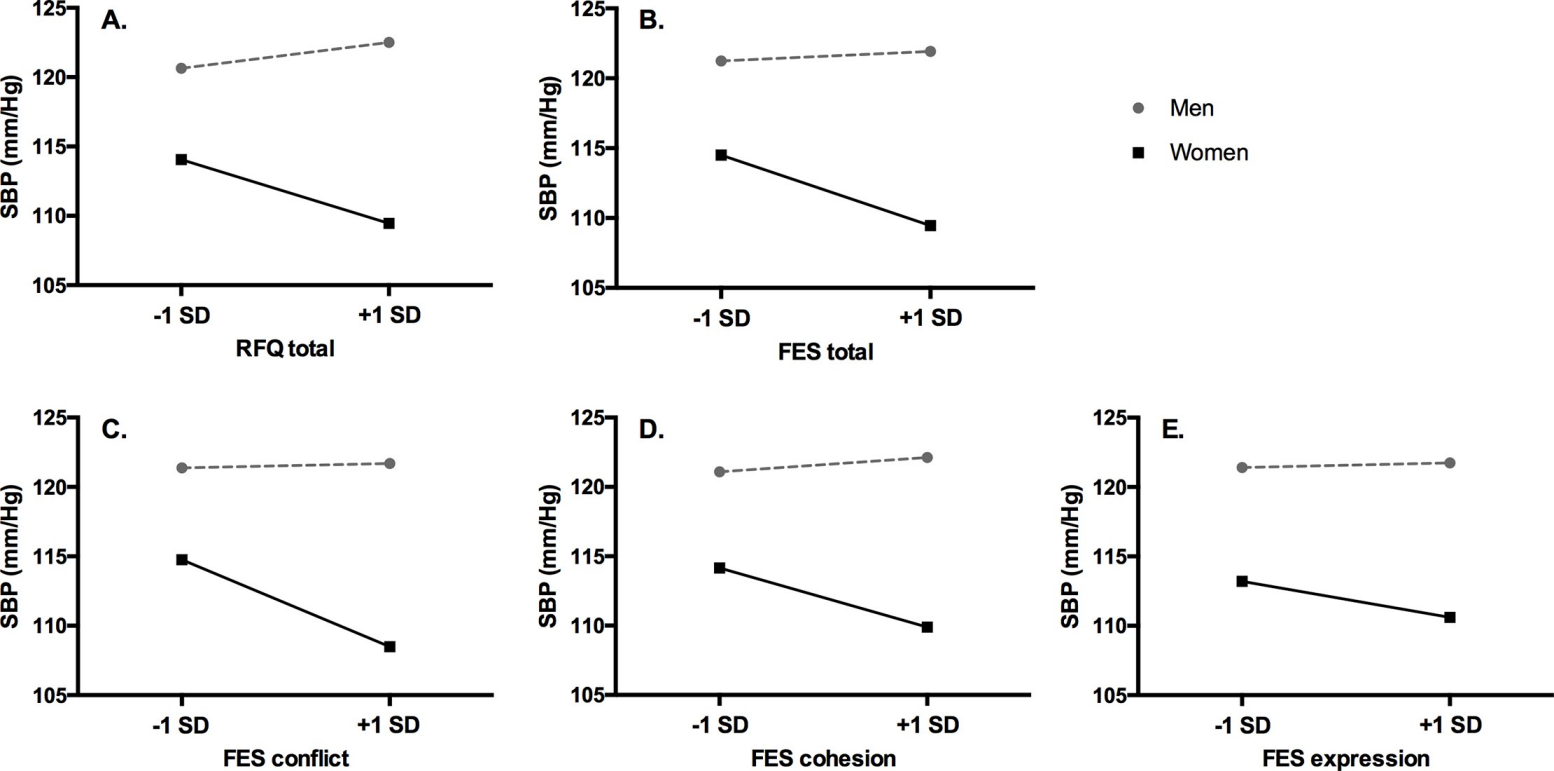
Participant sex moderates the association between adverse childhood family environments and resting SBP in adulthood. Indicators of childhood adversity are represented at +/- 1 standard deviation (SD). RFQ = Risky Families Questionnaire; FES = Family Environment Scale; SBP = systolic blood pressure.

**Fig 2:**
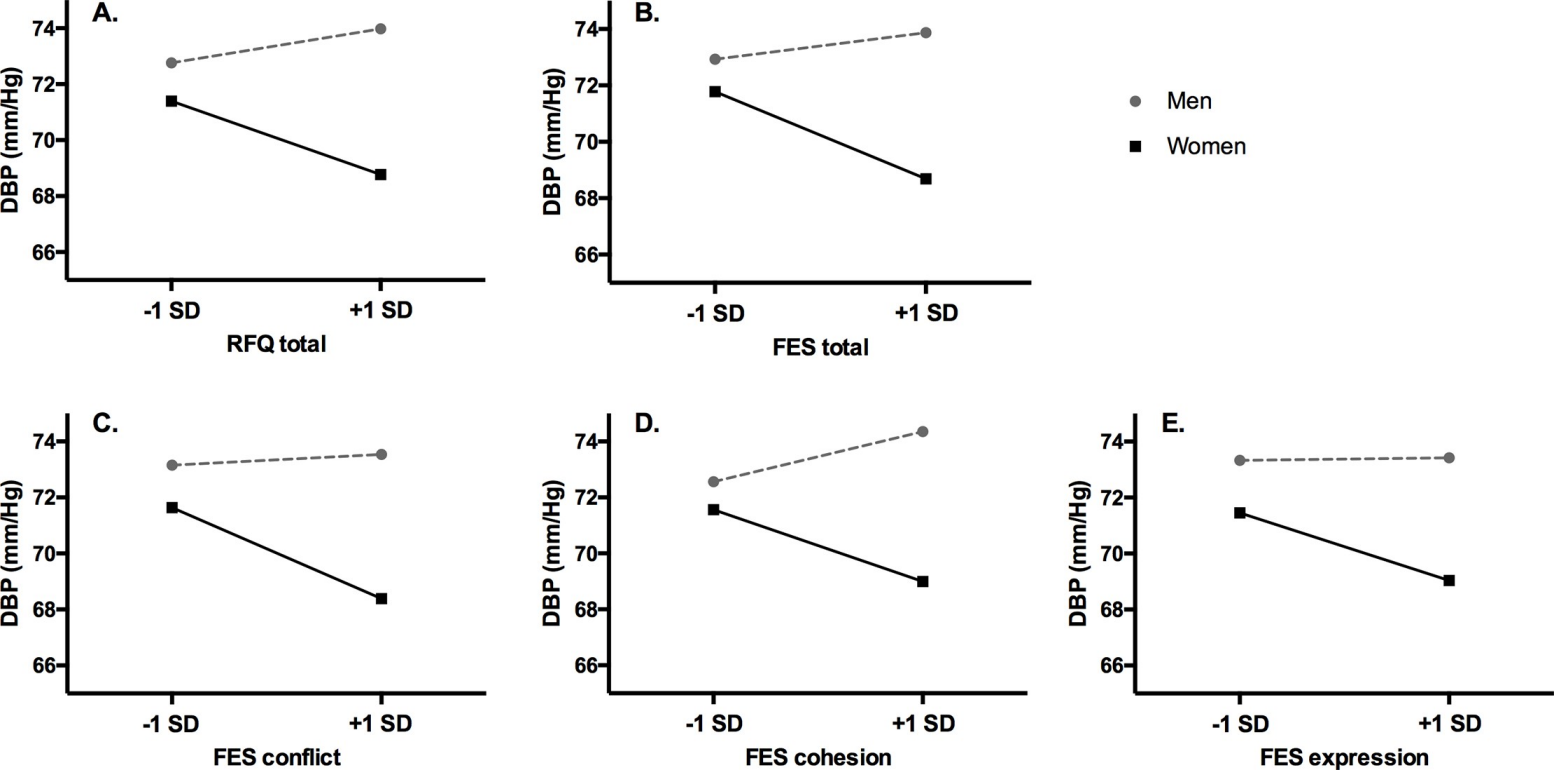
Participant sex moderates the association between adverse childhood family environments and resting DBP in adulthood. Indicators of childhood adversity are represented at +/- 1 standard deviation (SD). RFQ = Risky Families Questionnaire; FES = Family Environment Scale; DBP = diastolic blood pressure.

Sex interacted with several measures of adversity in participants’ childhood environments to predict adult physiological outcomes. Specifically, sex interacted with the RFQ and FES_total_ as well as the FES_conflict_ and FES_cohesion_ subscales to predict SBP (Fig 1). Similarly, sex interacted with the FES_total_ as well as the FES_cohesion_ subscale to predict DBP (Fig 2). Marginal effects on DBP were additionally found for the interactions between sex and the RFQ and FES_conflict_ subscale. Finally, participants’ HR was only predicted by the sex X FES_express_ interaction. Sex and early adversity did not interact to predict total salivary cortisol output. See Tables 2 and 3 for a detailed overview of these results. Notably, none of these results changed substantively when adding adult distress measures, with the one exception that the sex X FES_total_ interaction dropped to marginal significance (B = -.183; SE = .095; *p* = .056) in the saturated model for DBP.

**Table 2:** Multiple regression analyses examining moderation by sex of associations between early family environment and adult physiological outcomes.

	RFQ				FES_total_			
	B	β	SE	*p*	B	β	SE	*p*
Resting SBP (mmHg)								
Intercept	121.574		.901	< .001	121.587		.903	< .001
Childhood family environment	.091	.086	.082	.266	.031	.032	.079	.677
Gender (female)	-9.819	-.448	1.293	< .001	-9.605	-.438	1.295	< .001
Family environment x Sex	**-.316**	**-.201**	**.120**	**.009**	**-.274**	**-.178**	**.117**	**.021**
Overall model	R^2^ = .365; F(8,212) = 14.655; p < .001				R^2^ = .366; F(8,212) = 14.702; p < .001			
Resting DBP (mmHg)								
Intercept	73.371		.723	< .001	73.401		.723	< .001
Childhood family environment	.059	.080	.065	.367	.045	.062	.064	.479
Gender (female)	-3.289	-.215	1.037	.002	-3.168	-.207	1.037	.003
Family environment x Sex	-.187	-.170	.096	.054	**-.193**	**-.180**	**.094**	**.041**
Overall model	R^2^ = .166; F(8,212) = 5.062; p < .001				R^2^ = .170; F(8,212) = 5.215; p < .001			
Resting HR (bpm)								
Intercept	72.960		.979	< .001	73.001		.976	< .001
Childhood family environment	.045	.046	.089	.610	.043	.045	.086	.615
Gender (female)	3.062	.151	1.405	.031	3.223	.158	1.400	.022
Family environment x Sex	-.146	-.101	.131	.264	-.222	-.156	.127	.081
Overall model	R^2^ = .130; F(8,212) = 3.802; p < .001				R^2^ = .140; F(8,212) = 4.167; p < .001			
Cortisol AUC (nmol/l; log)								
Intercept	3.705		.022	< .001	3.703		.022	< .001
Childhood family environment	-.001	-.052	.002	.593	-.003	-.143	.002	.139
Gender (female)	-.070	-.160	.032	.033	-.067	-.154	.032	.040
Family environment x Sex	.001	.021	.003	.825	.003	.092	.003	.334
Overall model	R^2^ = .041; F(7,198) = 1.168; p = .323				R^2^ = .050; F(7,198) = 1.449; p = .188			

RFQ = Risky Families Questionnaire; FES = Family Environment Scale; SBP = systolic blood pressure; DBP = diastolic blood pressure; HR = heart rate; AUC = area under the curve; B = unstandardized beta weight; β = standardized beta weight. All analyses controlled for education, age, race (i.e., belonging to a racial minority or identifying as Caucasian), and birth control medications; BMI was also controlled when considering SBP, DBP, and HR. Sex (0 = male; 1 = female)

**Table 3:** Multiple regression analyses examining moderation by sex of associations between FES subscales and adult physiological outcomes.

	FES Conflict				FES Lack of Cohesion				FES Lack of Expressiveness			
	B	β	SE	*p*	B	β	SE	*p*	B	β	SE	*p*
Resting SBP (mmHg)												
Intercept	121.532		.891	< .001	121.603		.909	< .001	121.574		.917	< .001
FES subscale	.038	.015	.201	.849	.116	.047	.189	.542	.047	.015	.239	.844
Gender (female)	-9.906	-.452	1.281	< .001	-9.580	-.437	1.304	< .001	-9.671	-.442	1.321	< .001
FES subscale x Sex	**-.801**	**-.201**	**.297**	**.008**	**-.596**	**-.167**	**.277**	**.033**	-.422	-.089	.362	.245
Overall model	R^2^ = .377; F(8,212) = 15.444; *p* < .001				R^2^ = .360; F(8,212) = 14.334; *p* < .001				R^2^ = .347; F(8,212) = 13.572; *p* < .001			
Resting DBP (mmHg)												
Intercept	73.344		.721	< .001	73.457		.725	< .001	73.376		.728	< .001
FES subscale	.047	.025	.162	.774	.201	.118	.151	.185	.014	.006	.190	.942
Gender (female)	-3.333	-.217	1.035	.001	-3.178	-.207	1.040	.003	-3.130	-.204	1.049	.003
FES subscale x Sex	-.443	-.158	.240	.066	**-.489**	**-.196**	**.222**	**.028**	-.362	-.109	.288	.209
Overall model	R^2^ = .169; F(8,212) = 5.199; *p* < .001				R^2^ = .169; F(8,212) = 5.195; *p* < .001				R^2^ = .160; F(8,212) = 4.853; *p* < .001			
Resting HR (bpm)												
Intercept	72.939		.977	< .001	72.993		.981	< .001	73.098		.973	< .001
FES subscale	-.033	-.014	.220	.879	.082	.036	.204	.690	.294	.101	.254	.247
Gender (female)	3.008	.148	1.404	.033	3.258	.160	1.407	.022	3.250	.160	1.403	.021
FES subscale x Sex	-.301	-.081	.326	.357	-.452	-.137	.299	.132	**-.885**	**-.201**	**.384**	**.022**
Overall model	R^2^ = .132; F(8,212) = 3.878; *p* < .001				R^2^ = .137; F(8,212) = 4.038; *p* < .001				R^2^ = .147; F(8,212) = 4.398; *p* < .001			
Cortisol AUC (nmol/l; log)												
Intercept	3.706		.022	< .001	3.700		.022	< .001	3.703		.022	< .001
FES subscale	-.003	-.066	.005	.498	-.009	-.189	.005	.051	-.007	-.110	.006	.247
Gender (female)	-.069	-.159	.032	.034	-.066	-.152	.032	.042	-.065	-.149	.033	.049
FES subscale x Sex	.005	.062	.007	.509	.008	.117	.007	.224	.005	.053	.009	.577
Overall model	R^2^ = .042; F(7,198) = 1.201; *p* = .304				R^2^ = .059; F(7,198) = 1.696; *p* = .112				R^2^ = .047; F(7,198) = 1.331; *p* = .237			

FES = Family Environment Scale; SBP = systolic blood pressure; DBP = diastolic blood pressure; HR = heart rate; AUC = area under the curve; B = unstandardized beta weight; β = standardized beta weight. All analyses controlled for education, age, race (i.e., belonging to a racial minority or identifying as Caucasian), and birth control medications; BMI was also controlled when considering SBP, DBP, and HR. Sex (0 = male; 1 = female)

Next, separate models were fit for men and women to estimate simple slopes. Women who reported greater overall childhood adversity had lower SBP (RFQ: B = -.239; SE = .093; *p* = .012; FES_total_: B = -.259; SE = .089; *p* = .005) and lower DBP (FES_total_: B = -.154; SE = .065; *p* = .019) relative to women reporting less childhood adversity. Women who reported more conflictual (B = -.799; SE = .226; *p* = .001) and less cohesive (B = -.533; SE = .212; *p* = .014) childhood homes had lower SBP. Less cohesive childhood homes were marginally associated with lower DBP among women (B = -.302; SE = .154; *p* = .053). Finally, women who reported less expressive childhood homes had lower HR (B = -.572; SE = .252; *p* = .026). Results were largely unchanged when adding adult distress measures, with the exception of FES _total_ and FES_express_ no longer being associated with women’s DBP and HR, respectively (*ps* > .06). Associations among men were all in the opposite direction but none reached significance (*ps* > .20).

Additional, post-hoc sensitivity analyses adjusted for employment status, marital status and smoking status. This did not substantively alter results with the exception that the interaction between sex and FES_expressiveness_ on HR dropped to marginal significance (B = -.739, SE = .376, p = .051).

## Discussion

This study adds to the literature suggesting that childhood adversity is differentially associated with physiological outcomes among men and women later in adulthood. We found that, among women only, greater childhood adversity was associated with lower levels of baseline BP (although not HR and salivary cortisol). Importantly, these results persisted even after taking into account a wide range of concurrent psychosocial risk factors (e.g., anxious and depressed affect) and relevant demographic covariates (e.g., marital status and employment status). When considering particular aspects of the childhood home environment, more conflictual and less cohesive environments were more clearly associated with BP in women than was expressiveness within the home.

Given that early adversity is associated with greater disease risk, including CVD, among women, the directionality of the current findings is counterintuitive and counter to our predictions. However, these associations were generally consistent across both SBP and DBP and across different measures of childhood adversity. Our findings are also in line with a recent study reporting a negative association between the number of endorsed adverse childhood events (ACEs) and resting SBP in the laboratory among a generally healthy, all-female sample [27]. Similarly, Su et al. [28], reported higher baseline DBP (but not SBP) among male and female adolescents and young adults who had reported experiencing 2+ ACEs; moderation of these findings by sex was not investigated. Our current findings suggest that among a nonclinical sample of generally healthy women, greater reported exposure to moderate severity childhood adversity may be associated with the opposite pattern of basal cardiovascular hypoactivation, perhaps suggesting a down-regulated sympathetic nervous system (SNS) response among women exposed to childhood adversity although the extent to which the SNS is involved in longer-term BP regulation is still unclear [29]. Alternatively, our findings may suggest a survival advantage among women who, despite experiencing early adversity, were deemed to be generally healthy and thus may represent a particularly high-functioning subgroup of individuals.

Strengths of this study include the thorough assessment of resting BP (averaged across three measurement sessions over the course of one day in the laboratory), multiple indicators of the childhood home environment, and a generally healthy, non-clinical sample of adult men and women. Nonetheless, participants’ retrospective self-reports of their family home environment represents a limitation. Similarly, sex was self-reported; future research should tease apart the relative contributions of biological sex and socialized gender roles on the early adversity to BP connection. Lastly, this study was cross-sectional and the sample largely White. Thus, future research should investigate these associations in more diverse samples and examine potential mediating pathways, including individuals’ health behaviors and coping strategies, and broader psychosocial circumstances. Similarly, the fact that the sample was recruited via newspaper advertisements represents a potential limitation. Given participant demographic characteristics, in particular average years of education and rates of unemployment, it appears unlikely that this method of recruitment biased the sample towards more educated or wealthier individuals. Nonetheless, it is possible that focusing on recruiting via newspaper advertisements influenced the makeup of the sample in other ways. Finally, future research should investigate the possibility of three-way interaction effects between age, sex, and early adversity to inform our understanding of possible survivor effects if certain BP patterns are only apparent among older individuals, e.g., among older women exposed to greater early life adversity.

This study suggests that reporting exposure to moderate severity childhood adversity is associated with lower resting BP among healthy women, but not men, with potential implications for their long-term health. This is counter to some findings from clinical samples and further highlights the importance of separately considering effects of the early family environment on physiological health outcomes among men and women.
